# Biological interactions of biocompatible and water-dispersed MoS_2_ nanosheets with bacteria and human cells

**DOI:** 10.1038/s41598-018-34679-y

**Published:** 2018-11-06

**Authors:** Jasneet Kaur, Manjot Singh, Carmela Dell‘Aversana, Rosaria Benedetti, Paola Giardina, Manuela Rossi, Mohammadhassan Valadan, Alessandro Vergara, Anna Cutarelli, Angela Michela Immacolata Montone, Lucia Altucci, Federica Corrado, Angela Nebbioso, Carlo Altucci

**Affiliations:** 1 Department of Physics, “Ettore Pancini”, University of Naples “Federico II”, Naples, Italy; 2 Department of Chemical Sciences, University of Naples “Federico II”, Naples, Italy; 3 Department of Precision Medicine, University of Campania “L Vanvitelli, Vico L. De Crecchio” 7, 80138 Naples, Italy; 4 Department of Earth, Environment and Resources Sciences, University of Naples “Federico II”, Naples, Italy; 5 Experimental Zooprophylactic Institute of Southern Italy, Portici, Italy

## Abstract

Two dimensional materials beyond graphene such as MoS_2_ and WS_2_ are novel and interesting class of materials whose unique physico-chemical properties can be exploited in applications ranging from leading edge nanoelectronics to the frontiers between biomedicine and biotechnology. To unravel the potential of TMD crystals in biomedicine, control over their production through green and scalable routes in biocompatible solvents is critically important. Furthermore, considering multiple applications of eco-friendly 2D dispersions and their potential impact onto live matter, their toxicity and antimicrobial activity still remain an open issue. Herein, we focus on the current demands of 2D TMDs and produce high-quality, few-layered and defect-free MoS_2_ nanosheets, exfoliated and dispersed in pure water, stabilized up to three weeks. Hence, we studied the impact of this material on human cells by investigating its interactions with three cell lines: two tumoral, MCF7 (breast cancer) and U937 (leukemia), and one normal, HaCaT (epithelium). We observed novel and intriguing results, exhibiting evident cytotoxic effect induced in the tumor cell lines, absent in the normal cells in the tested conditions. The antibacterial action of MoS_2_ nanosheets is then investigated against a very dangerous gram negative bacterium, such as two types of *Salmonellas*: ATCC 14028 and wild-type *Salmonella typhimurium*. Additionally, concentration and layer-dependent modulation of cytotoxic effect is found both on human cells and *Salmonellas*.

## Introduction

The advent of an extremely interesting novel class of two dimensional materials (2DMs) was triggered by the successful isolation of single atomic layers of graphene. The unprecedented properties of graphene sparked a search for additional 2D materials with their own unique characteristics^1^. In general, 2DMs possess large surface areas combined with outstanding electronic, optical, electrochemical, mechanical and thermal properties that are opening new channels for fundamental scientific research and advanced technological applications^2,3^, there including sensing, catalysis, energy storage and functional nano composites^4,5^. The feature of being some of the thinnest 2D structures among all known materials with very high specific surface area makes them indispensable for applications requiring high levels of surface interactions at the nanoscale i.e. into the frontiers between biology and medicine such as antibacterial activity^6,7^, biosensors^8,9^, drug delivery^10,11^, cancer cell diagnosis and cell imaging^12^.

To exploit the full potential of 2D TMD nanosheets in applications, control over their production is very crucial. Therefore, the need for more versatile and scalable techniques for 2DMs exfoliation is apparent. After the era of mechanical exfoliation, many serious efforts have been made to adopt new techniques to produce 2D nanosheets which includes chemical vapour deposition^13^ and exfoliation in different liquids or solutions^14^ such as chemical oxidation followed by reduction^15,16^, electrochemical exfoliation^17,18^, ion intercalation^19,20^ and liquid phase exfoliation (LPE)^21,22^.

Among the above cited techniques, LPE is the most versatile, scalable and cost effective technique for the production of few-layer nanosheets (1–10 stacked monolayers), with low monolayer content^23,24^. Particularly in this technique, a careful optimization of exfoliation parameters such as, choice of green solvents, initial concentration of the solution, exfoliation time and controlled centrifugation for size and thickness selection of 2D nanosheets is very crucial to understand their environmental impact and behaviour in biological media^21,25–27^. Organic solvents such as ethylene glycol, Methyl-2-Pyrolidone and Iso-propyl Alcohol are substantially used for the large scale production of high monolayer content 2D nanosheets^25,28^. But their chemical fate and toxicological behaviour strongly limits their use for biomedical research onto live matter.

Therefore, water dispersed, defect free and biocompatible 2D MoS_2_ nanosheets are absolutely needed in place of nanosheets exfoliated in organic solvents. By the way, exfoliation of 2D nanosheets in water is a big challenge itself^25,29–31^. For example, Ma *et al*. reported the cavitation induced exfoliation protocol of MoS_2_ dispersion and its consequences based on Hemi-Wicking model^32^.

Toxicity, environmental impact and biocompatibility of MoS_2_ onto different human cell lines is very important to investigate, in view of the more and more massive use of these 2D materials in a number of practical applications and their increased presence in the human day-to-day life. Morphology, size and thickness of 2D nanosheets are some key parameters to induce and potentially control the surface interactions of MoS_2_ nanosheets onto live matter. Functionalized MoS_2_ nanosheets have been studied with different human cell lines to tap the potential of 2D nanosheets in various biomedical applications such as drug delivery, cancer diagnosis and cell imaging^33–35^. For instance, Coleman and co-workers reported the size and concentration dependent toxicity of MoS_2_ nanosheets on three different established cell lines^36^. Siepi *et al*. reported on the biocompatible lysozyme-functionalized exfoliated MoS_2_ nanosheets. They incubated MoS_2_ nanosheets on two different cell lines (HeLa and HaCaT) with no cytotoxicity evidence at higher concentrations^37^. J. H. Appel *et al*. reported on the interactions of naked MoS_2_ nanosheets, obtained by mechanical exfoliation and chemical vapor deposition, with human epithelial kidney cells (HEK293f) observing low cytotoxicity and genotoxicity in their experimental conditions^38^. P. Shah *et al*. probed the effect of MoS_2_ nanosheets produced by liquid exfoliation onto rat cells finding a relatively good biocompatibility at high 2D material concentration^39^.

The wide area of biomedical applications of MoS_2_ nanosheets also embraces its potential to exert antibacterial effects against various pathogens through induction of physical damage and oxidative stress which leads to continuous disruption of bacterial cells and eventually to cell death^40–43^. These promising results have attracted MoS_2_ nanosheets as a potential candidate, better than graphene and its derivatives, for significant antibacterial applications. Concentration dependent studies of MoS_2_ nanosheets with *Escherichia coli*^44^, *Bacillus subtilis* and *Staphylococcus aureus*^42^ revealed a decrease in the bacterial survival rate with increase in dispersion concentration. On the other hand, *Salmonella typhimurium (S. typhimurium)*, a discretionary gastric pathogen which is responsible for food poisoning in humans resulting in gastroentertitis^45^ and represents a very dangerous gram negative bacterium, was studied only quite recently until now in its interaction with bare MoS_2_ nanosheets^38,42,46–48^.

Therefore a thorough study on the different interaction pathways of naked MoS_2_ nanosheets with different human cell lines and various pathogens is highly needed and still missing in the most important case, namely for nanosheets produced by eco-friendly methods and dispersed into water based media that is the native context of biological matter.

To this aim, the present research is focused on the noticeable progress on green and scalable production of MoS_2_ nanosheets in water as a pure solvent, having stability up to three weeks by carefully optimizing critical exfoliation parameters^26^. Such a long stability time in water solvent, which is a non-trivial result, is crucial to test the impact of 2DMs with biological live matter in its native context, as experiments aimed at these goals may take a few days or even longer to be completed. Thus, we stress that our innovative preparation of naked MoS_2_ nanosheets in water solvent represents an essential step ahead for an appropriate characterization of 2DM - live matter interactions in its natural environment. Biological interactions of bare MoS_2_ nanosheets are investigated with three different kinds of human cells, two tumoral, MCF7 (breast cancer) and U937 (leukemia), and one normal, HaCaT (epithelium), and two different kinds of *Salmonella*- ATCC 14028 and wild type *S.typhimurium*. It is worth noting that while MCF7^49–52^, and HaCaT^37,53^ cells have been already partly checked in their interactions with MoS_2_ nanosheets, U937-MoS_2_ interactions are completely unknown so far. Yet, MCF7 (Breast Cancer), Hela (Human Cervical Cancer), PC3 (Human Prostate Cancer), SMCC-7721 (Human Hepatocellular Carcinoma), B16 (Mouse Melanoma) and A549 (Human Lung Carcinoma) as cancer cell lines have been also recently tested as models for the interactions between human cells and 2D functionalized nanomaterials of various kind, there including 2D Black Phosphorus nanosheets^54^, 2D Boron nanosheets^55^ 2D Antimonene quantum dots^56^ 2D Antimonene nanosheets^57^ and Tin Sulfide nanosheets^58^. It is worth stressing, by the way, that in each of these cases the 2DMs, nanosheets and also quantum dots in one case^56^, are functionalized by forming a nano material-Polyethylene glycol (PEG) complex to which the specific Doxorubicin anti-cancer drug is added, differently from what studied in our experiments, where the interactions between living cells and naked nanoflakes are probed. As a matter of fact, in these cases the scheme of the interaction between 2DMs and cells is basically different from our case, since the authors induce a cytotoxic effect of the nano-complex by photo-activation of the material due to illumination with near-infra red light (780–808 nm wavelength). The near-infra red light is efficiently absorbed by the nano-complex (2DM-PEG-Anticancer drug) and this induces a heat of the whole environment and a change in the pH in the neighbour of the nano-complex that causes the efficient interaction of the anti-cancer drug with the cells. Therefore, while these studies have been focused on specific application of 2DMs mainly as a support for a photo-active complex containing also PEG and anti-cancer drug, resulting in an induced cytotoxic action useful in theranostics and therapeutics, our paper reports on the non-mediated interaction of bare nanoflakes with human cells of various types and possible applications due to the intrinsic observed cytotoxic action.

Diagnostics of the nanosheets is carried out based on several well established methods, such as Raman^59,60^ and UV-Vis absorption spectroscopy^22^ and *ζ*- Potential^31^. Antibacterial and cell viability studies were analyzed via Methylthiazolyldiphenyl-tetrazolium bromide (MTT) test to quantify the cell/bacterial viability at a given concentration. We found a very interesting and novel result: while tumoral cells exhibited an unexpected and strong reaction to MoS_2_ nanosheet treatment in the tested conditions, leading to cell death, even more striking in U937 than in MCF7, no appreciable impact was observed on normal cells by nanosheets, even on the long term time scale of 96 h after treatment. SEM analysis was also performed to study the change in morphology of human cells and *S.typhimurium*. Colony counting images revealed an evident antibacterial effect even at low concentration of MoS_2_ nanosheets dispersion.

## Results

### Dispersion stability of water exfoliated MoS_2_ nanosheets

It is worth stressing that a number of parameters are vital for the success of green route exfoliation of MoS_2_, such as initial concentration of the dispersion, shape of the glass tube and of the probe (flat head or narrow cone shaped), sonication time, amplitude of the sonicator signal, centrifugation time and speed. Among these parameters, the concept of ‘dead zones’ as explained by J. L. Capelo *et al*. is of paramount importance to have a minimum distance between the probe and the bottom of the tube used for exfoliation. The larger the contact area of the probe with the material the more effective the exfoliation and the transfer of acoustic energy and ultrasonic intensity through the probe. Rest of the parameters play a crucial role for high quality and highly stabilized nanosheets dispersion.

In our case, the exfoliation of bulk MoS_2_ powder was performed in elix water (as a pure solvent) using a tip sonicator for 3 h at 35 W. After optimizing a range of initial concentrations and various exfoliation constraints, a stable dispersion for up to three weeks was achieved. This was in good consideration to utilize this material for biological applications without the use of any organic solvent or any stabilizer. Further experimntal details are reported in Materials and Methods section.

### UV-visible extinction spectroscopy

**Figure 1:**
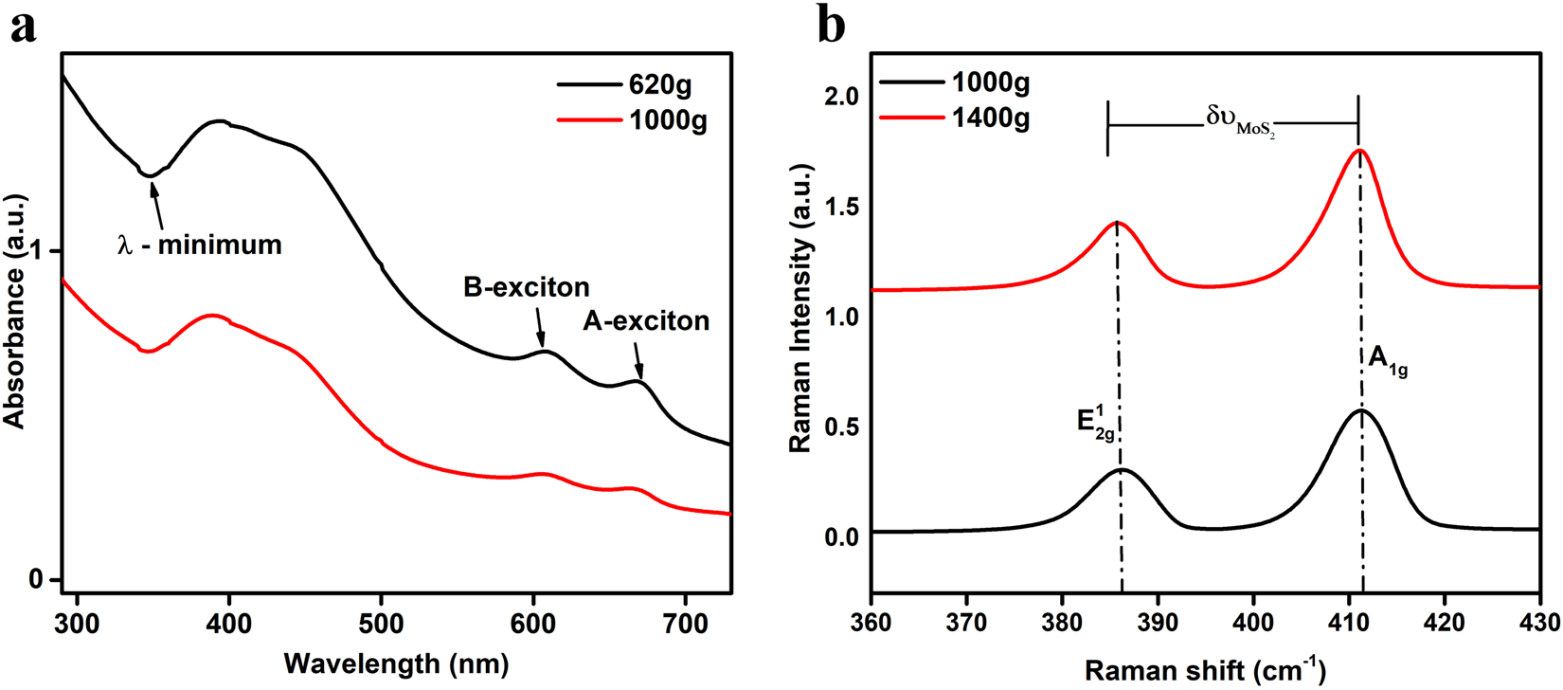
Material characterization. (**a**) UV-Visible extinction spectra of 2D MoS_2_ nanosheets dispersion at 620 g and 1000 g. (**b**) Raman spectra of the two main vibrational modes ${\nu }_{2g}^{1}$ and *A*_1*g*_ of MoS_2_ nanosheets centrifuged at 1000 g and 1400 g. Raman shift in the region from 380–412 *cm*^−1^ range represents MoS_2_ nanoflakes in the range from 2–4 layers.

In case of 2DMs, liquid processing of bulk materials via the green route of production results in few layered MoS_2_ nanosheets. Therefore, UV-Visible spectroscopy is a very basic measurement technique in general to extract the useful information from such colloidal dispersions. The extinction spectra in the UV-visible region of MoS_2_ samples contain the contribution from both absorbance and scattering components. Both of these components are size dependent. In our experiment protocol, at lower centrifugal forces 40 g and 160 g, scattering component was more dominant with high extinction peaks at 750–800 nm. Whereas, at higher centrifugal forces 620 g and 1000 g, A-exciton peak shifted towards the lower wavelength region. With the increase in centrifugal force, number of layers per flake decreases which results in few layered enriched dispersions. The extinction spectra of MoS_2_ after the final steps of centrifugation at 620 g and 1000 g are shown in Fig. 1a. The physical parameters of MoS_2_ dispersion at different centrifugal forces are shown in Supplementary Table S1. Extinction parameters based on the formulation^21^ obtained immediately after the exfoliation of MoS_2_ nanosheets and after three weeks of storage are shown in Supplementary Table S2.

### *ζ*- Potential Measurements

Generation of surface charges over the surface of 2D nanosheets plays a crucial role to understand the stability of liquid exfoliated dispersions. To identify these surface charges, electrophoretic mobility measurements (*μ*) are performed in general. These (*μ*) measurement works as a quantifying tool to understand the electrostatic stabilization between the nanosheets by estimating the zeta potential (*ζ*). In case of 2DMs, dynamic interactions among the nanosheets and their electrostatic stabilization play a fundamental role to anticipate the stability of liquid dispersions. It was observed that after the exfoliation, the MoS_2_ flakes exhibit high surface charge density depending upon the different centrifugal forces applied as seen from the Table 1. The experimental details of *ζ*-potential measurement are explained in Supplementary file.

**Table 1:** *ζ*- Potential values of MoS_2_ nanosheets dispersion at different centrifugal forces.

Centrifugal force g	Zeta potential (*ζ*) mV	electrophoretic mobility (*μ*) *μ*
620	−23.9 ± 0.6	−1.88 ± 0.04
1000	−29.2 ± 1.3	−2.9 ± 0.1

### Raman Micro-spectroscopy of MoS_2_ nanosheets in absence of cells

Raman spectroscopy is a widely employed tool to estimate the thickness of TMD nanoflakes^21,59–61^. The Raman spectrum of MoS_2_ shows characteristic bands, ${E}_{2g}^{1}$ and *A*_1*g*_, corresponding to in-plane and out-of-plane vibrational modes, that for bulk fall at about 380 *cm*^−1^ and 403 *cm*^−1^ respectively^59^. MoS_2_ nanostructuring modifies the Raman features of the bulk with an increase for the ${E}_{2g}^{1}$ frequency and a corresponding decrease of the *A*_1*g*_.

1 $${\rm{\Delta }}{\nu }_{Mo{S}_{2}}={\nu }_{{A}_{1g}^{-}}{\nu }_{2g}^{1}$$

The frequency shift allows for an identification of the number of layers in the nanoflakes^59,60^. In Fig. 1b, Raman spectra of MoS_2_ nanoflakes in absence of cells centrifuged at 1000 g and 1400 g (used to exfoliate MoS_2_ in nanosheets) are shown, with laser excitation at 514.5 nm. We observed similar modification in the Raman spectrum compared to bulk for both centrifugal protocols, with a common range of frequency shift ${\rm{\Delta }}{\nu }_{Mo{S}_{2}}$ of peaks ranging in the 23–24.6 *cm*^−1^ window. The ${\rm{\Delta }}{\nu }_{Mo{S}_{2}}$ range observed via Raman micro-spectroscopy corresponds to a nanostructuring spanning from 4 to 2 layers. These micro-Raman spectroscopy results look consistent with the range of nanostructuring indicated by UV-vis absorption.

### Cytotoxicity study on different cell lines

Cytotoxicity experiments were performed using two cancer cell lines (U937 and MCF7) and one non-cancer cell line (HaCaT).

### Effects of 2D MoS_2_ nanosheets on cellular growth

Cytotoxicity experiments were performed in two different cell culture conditions: in suspension and in adhesion. This approach allowed to examine the interaction between MoS_2_ nanosheets and cells under the condition in which bona fide either the entire cell surface - in case of suspension cultures - or part of it - cell monolayer of adherent cultures - resulted exposed to 2D nanomaterial. The impact of different concentrations ($\bar{C}$), mean number of layers ($\bar{N}$) and mean lateral size ($\bar{L}$) of MoS_2_ nanosheets dispersion was investigated upon their incubation with two tumoral cell lines (U937 and MCF7) and one normal cell line (HaCaT), as shown in Supplementary Table S3. It is worth noting that for cells the effective concentration of the nanomaterial is lowered by about a factor of four as compared to the concentration value of the initial preparation.

**Figure 2:**
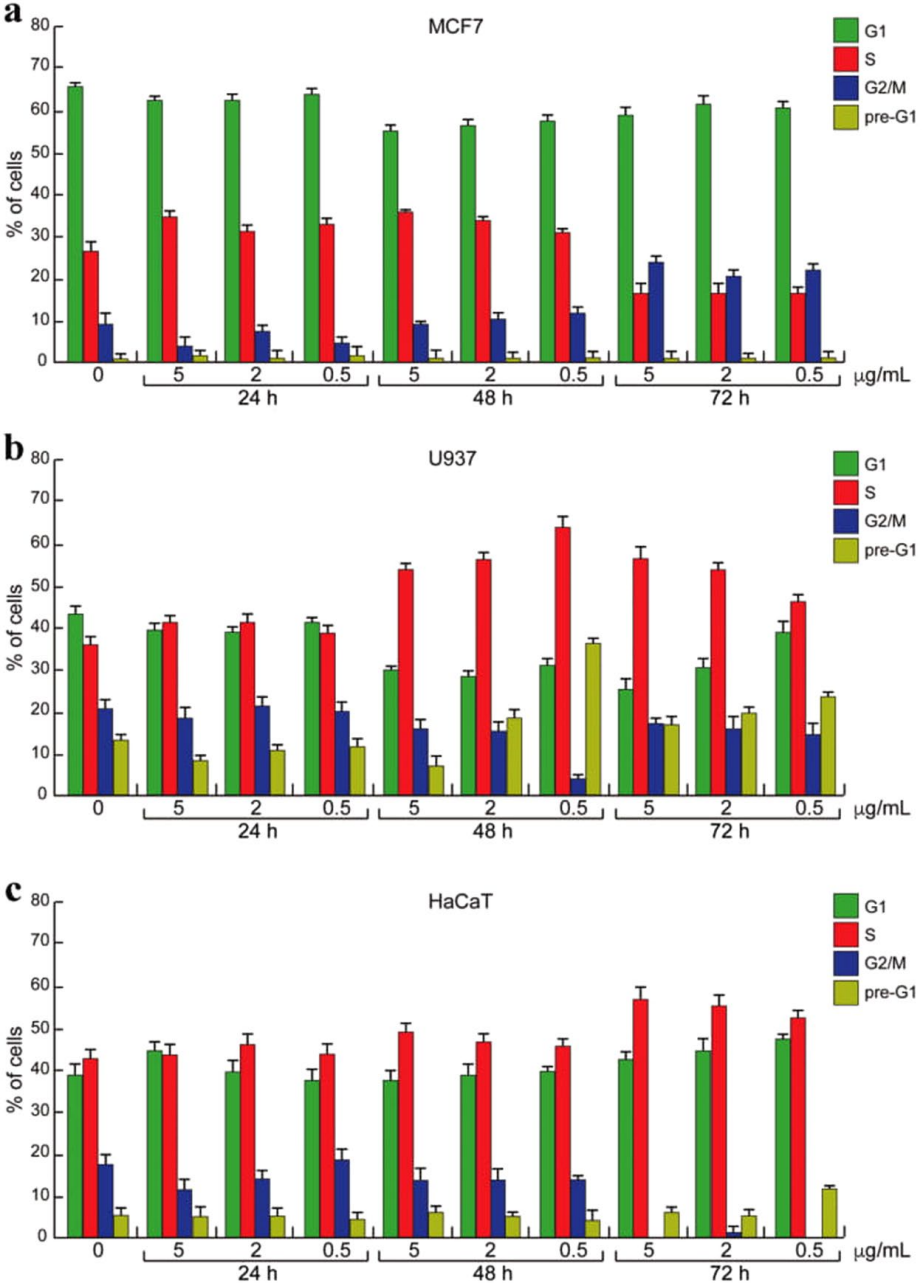
Cell cycle analysis. (**a**) MCF 7, (**b**) U937 and (**c**) HaCaT cell lines at different final concentrations of 2D MoS_2_ dispersion after 24 h, 48 h and 72 hours from the beginning of the treatment. Error bars indicate standard deviation of triplicate analysis.

The reason is, to avoid an excessive dilution of the culture media and nutrients therein. The dispersion was drop cast into the cell medium and the incubation was carried out for 24, 48 and 72 h. At the end of incubation, the cell cycle progression was determined by flow cytometer or fluorescence activated cell sorting (FACS), a technique that discriminates the cells at different phases of cell cycle for their content in DNA (Fig. 2). The cytometer processes the fluorescence intensity of a group of cells labeled with fluorescent dye (PI) that is able to bind DNA. The data is displayed as number of cells versus fluorescence intensity, a number proportional to cell DNA content. The cell scattering shows two peaks: G1 (gap 1), the gap between mitosis (nuclear division) and DNA replication, corresponding to cells metabolically active but that do not replicate their DNA and G2 (gap 2) corresponding to cells that grows and synthesize the proteins for mitosis. Between G1 and G2 there is the S phase in which the cells replicate their DNA. The cell death is revealed as percentage of cells in pre-G1 phase, corresponding to a pick of fragmented DNA^62,63^.

Our data of Fig. 2a revealed that MoS_2_ dispersion did not affect MCF7 distribution along cell cycle phases within 48 hours even at the highest concentration. Only the longest incubation time was able to induce a decrease of S phase in dose-independent manner and increase in the progression to G2/M phase (Fig. 2a). Conversely, cell cycle distribution of U937 cell was strongly affected in time dependent manner (Fig. 2b). At 48 h, MoS_2_ dispersion at three different concentrations was able to induce a block in S phase and an increase of cell death percentage. Notably, the lowest used concentration of MoS_2_ dispersion (0.5 *μg*/*mL*) induced the strongest effect on cell death after 48 h. However, the cell death (pre-G1) was attenuated at 72 h probably because of sedimentation of 2D MoS_2_ dispersion. Similar trends were found in HaCaT cells as shown in Fig. 2c. In human keratinocyte cell line, the induction of both S-phase block and cell death were observed at 72 h. Delayed response on cell cycle progression accounted for the slower proliferation rate of this normal cellular model.

### Effects of 2D MoS_2_ nanosheets on cellular viability

**Figure 3:**
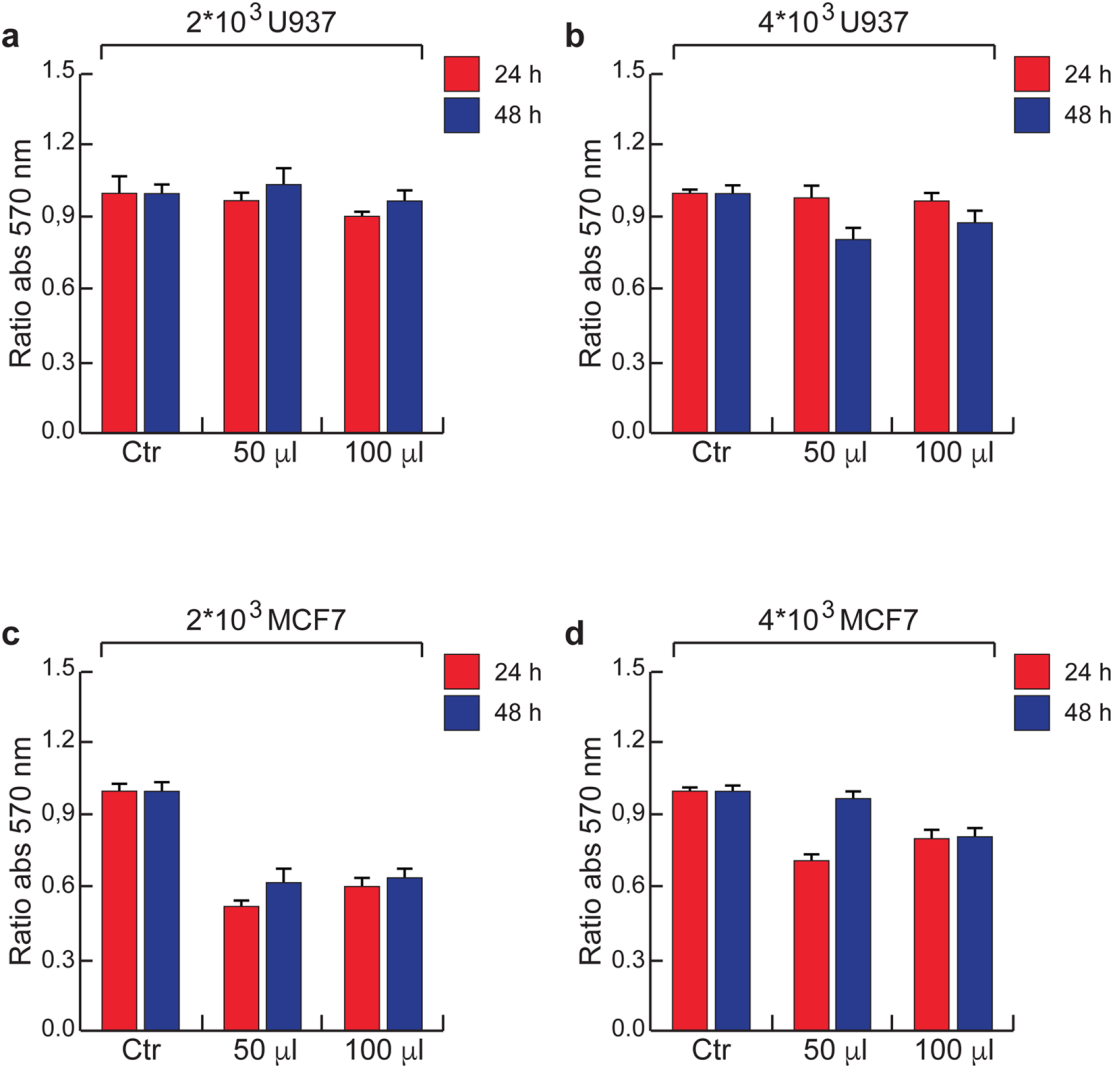
MTT analysis. (**a**,**b**) MTT assay performed on U937 cell line with (a) 2000 and (b) 4000 cells at 570 nm absorbance for 24 h and 48 h. (**c**,**d**) MTT assay performed on MCF7 cell line with (**c**) 2000 and (**d**) 4000 cells at 570 nm absorbance for 24 h and 48 h. Error bars indicate three independent experiments in (**a**–**d**).

To better investigate the cytotoxicity, viability of the above mentioned three human cell lines was investigated after 24 and 48 h of exposure to MoS_2_ dispersion. MTT experiments were performed by growing cells onto plates coated with 50 and 100 *μL* drops of MoS_2_ nanosheets. The exfoliation of MoS_2_ dispersion exhibit 14 *μg*/*mL* $\bar{C}$ with 6 $\bar{N}$ and $\bar{L}$ of 220 nm. The viability of U937 cells was not affected (Fig. 3a) by the presence of MoS_2_ nanosheets at the bottom of the cell plate even at the highest cell density (Fig. 3b). In fact, differently from what we observed for U937 cells, MTT analysis performed on the adherent MCF7 cells showed an interesting interference effect induced by 2D nanosheets as shown in Fig. 3(c,d). The presence of MoS_2_ nanosheets coating on the plate mainly affected the viability (and/or adhesive properties) of the cells. After 24 h more than 50% of cell death was already observed (Fig. 3c). No significant effect of both the quantity of MoS_2_ nanosheets and cellular concentration was noted. In Fig. 3d, taking twice the number of MCF7 cells present in 3c the cell viability in this case was much less affected than in 3c. We ascribe this finding to the specific type of interaction between the adhered MCF7 cells and the nanoflakes. In fact, in case of adhered cells the interaction always takes place through the interface surface between the cell medium and the nanoflakes. This interface, constituted by the most external cell layer, is approximately keeping the same size and involving the same number of cells regardless the actual entire volume of the growth cells below the separation surface. Therefore, simply increasing the number of cells while keeping the same interface results in minimizing the interaction between adhered cells and MoS_2_ nanosheets.

Similar effects were observed in HaCaT cells incubated with MoS_2_ nanosheets coated onto the plates (Properties of Exfoliated MoS_2_ nanosheets dispersion are shown in Supplementary Table S4). Like MCF7, HaCaT cell line viability was strongly affected by the presence of MoS_2_ nanosheets coated over the plates. Here we can also see that in the case of 14 *μg*/*mL* $\bar{C}$, but with two different $\bar{N}$, 6 and 10, the induced evident reduction of cell viability is not changing, suggesting that no major role is played in such a case by the $\bar{C}$. The above findings corroborated the idea that a prolonged physical proximity between 2D nanosheets and cells is required to induce the cell death. In fact, while this is certainly the case for MCF7 and HaCaT that grow up in adhesion, for U937 that grow up in suspension MoS_2_ nanosheets can be much more diluted in the entire volume of the solution so to interact much more weakly with cells. Normally the interaction of nanomaterials with cells is studied with the latter being placed at the bottom of a culture plate. *In vitro*, cellular response to nanoflakes can also be evidently influenced by the altered diffusion and sedimentation velocities of the nanostructured flakes^64^, as well as by electrostatic forces arising from the interaction between exfoliated MoS_2_ nanosheets that are negatively charged (Table 1) and cell membrane which is typically positively charged on the external side, thus generating a negative transmembrane potential^65^. Within the electrostatic forces that may play a role in the interactions between cells and MoS_2_ nanosheets it is worth mentioning also those driving the ion channels regulation through the cell membrane, as in case of potassium ion, that might be affected by the presence of 2DM nanosheets^66^.

Cell adhesion is an important aspect in cell proliferation, and can play a role in the interaction of cells incubated with nanomaterials. Typically, cells are prepared so to adhere before the addition of the nanomaterial solution to the preparation^67^. In our case, cells are adhered onto the plates coated with two MoS_2_ dispersion drops having different volumes. Since U937 cells by nature grow up in suspension, typically tend to a minor surface-like interactions resulting in a poor adhesion and thus a weaker interaction with the nanoflakes. Therefore, the cell viability was not affected as in the case of the other cell lines. On the other hand, both MCF7 and HaCaT cells grow up in adhesion to a surface. This property makes them more appropriate for surface-like interactions, there including those ones with the nanomaterial. This leads MCF7 and HaCaT cells to exhibit a strong decrease in cell viability when incubated with 2D MoS_2_ nanosheets, in the range of ≈0–50% in our experimental conditions.

### MTT assay on HaCaT and MCF7 cells pre-incubated with MoS_2_ nanosheets

Walking on this line, we exploited another interaction pathway of pre-incubation and rotation of 2D nanosheets with cells, so to increase their direct contact of interaction in the medium. In this setting, adherent HaCaT cell line was pre-incubated for 1 h in gentle rotation with the indicated quantities of MoS_2_ nanosheets and then cultured for next 24 and 48 h in standard growing conditions. In this condition, cells viability was profoundly impaired in the presence of MoS_2_ nanosheets as shown in Supplementary Table S4. Our data also revealed that cellular response was somehow dependent on $\bar{C}$ and $\bar{N}$ of MoS_2_ nanosheets, though the mechanism for interplay between $\bar{N}$ and $\bar{C}$ on the induced biological effects in this peculiar geometry of interaction still needs to be more deeply investigated. The general behaviour here indicates that adding MoS_2_ nanosheets in the preparation as described above leads always to a strong cell viability decrease, even larger than 65%, and suggests to some extent that the lower the concentration the higher the cell viability decrease.

In Supplementary Table S5 we report the viability of MCF7 cells via MTT assay, pre-incubated with 2D MoS_2_ nanosheets having different $\bar{C}$ and $\bar{L}$. Even in this case there is a strong impact of the nanosheets over the cell viability, even stronger than in HaCaT, in the same conditions. The sample absorbance decreases in this case from 1 (negative control) to a value in the 0.1–0.35 range, when checked at 24 h and 48 h after treatment. No clear dependence is observed in this case on the $\bar{C}$ and on the parameters characterizing the nanosheets such as $\bar{N}$ and $\bar{L}$.

### Cell morphology by scanning electron microscope (SEM)

To better characterize the microscopic structural features of the interaction between cells and MoS_2_ nanosheets, SEM experiments were performed. After the pre-incubation for 1 h with MoS_2_ dispersion, the three cell lines used were conventionally cultured on converglasses. Then, cells were observed by SEM after 24 h of incubation with two dispersions of MoS_2_ nanosheets (having 10 *μg*/*mL* $\bar{C}$ with 6 $\bar{N}$, and 14 *μg*/*mL* $\bar{C}$ with 3 $\bar{N}$). SEM images clearly revealed the deposition of some MoS_2_ flakes over the cell surface.

**Figure 4:**
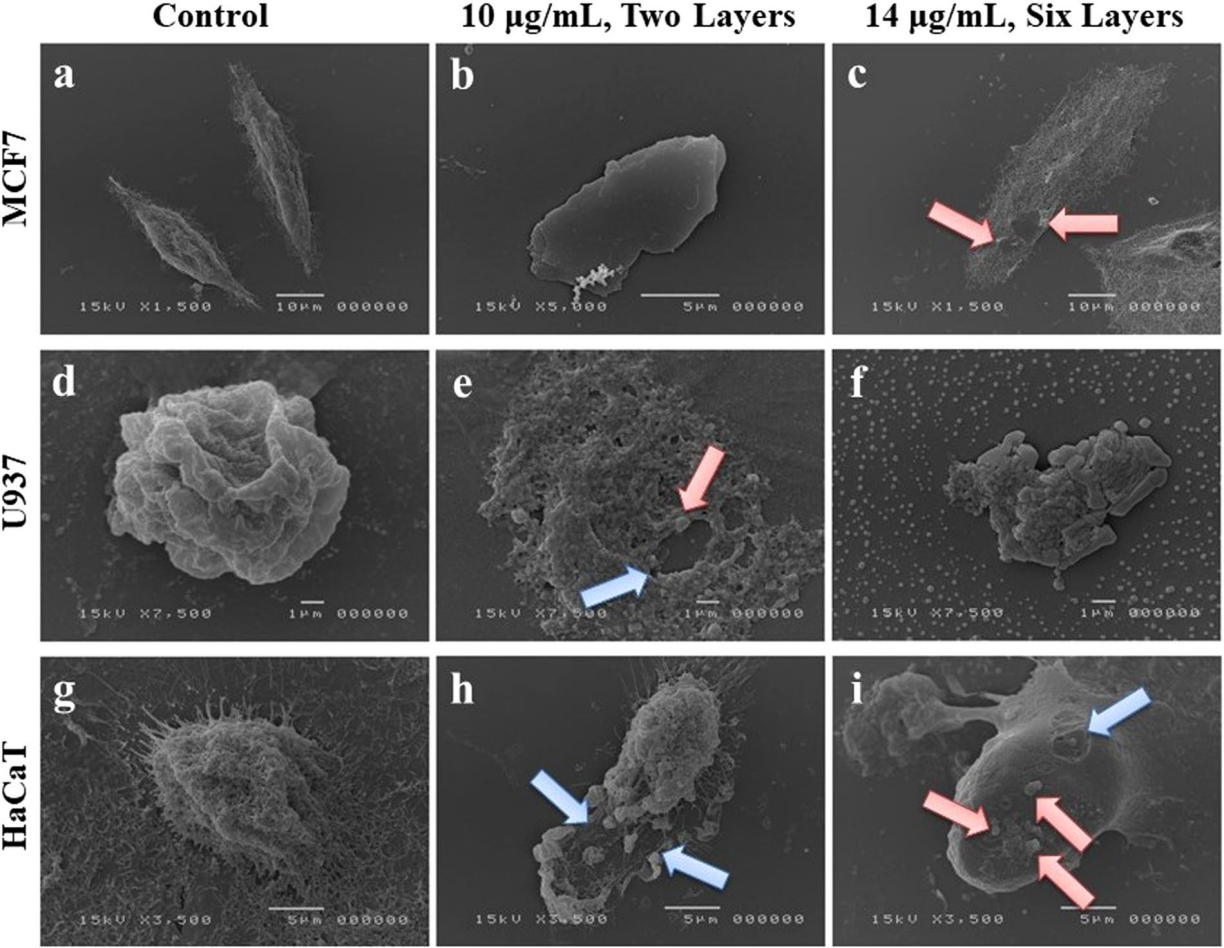
SEM analysis of human cell lines. (**a**–**c**) MCF7 (top, the red arrows indicating the nanoflakes onto the cell), (**d**–**f**) U937 (middle, the blue arrows indicating the damaged areas and the red the nanoflakes) and (**g**–**i**) HaCaT cells (bottom, the blue arrows indicating the damaged areas and the red the nanoflakes) untreated and treated with MoS_2_ dispersion at the indicated concentrations. The $\bar{N}$ was two at 10 *μg*/*mL* and six at 14 *μg*/*mL*.

Figure 4 shows the interaction of MoS_2_ nanoflakes with MCF7, U937 and HaCaT cell lines, the flakes having $\bar{L}$ in the 0.5–10 *μ*m range. MCF7 line of Fig. 4a represents the negative control case (absence of MoS_2_ dispersion) with two MCF7 cells exhibiting their typical epithelial morphology. MoS_2_ flakes from the two samples (10 *μg*/*mL* and 14 *μg*/*mL*) were added, 4b showing the typical structural aspect of a flake. The addition of the MoS_2_ nanoflakes resulted in an alteration of the cell structure that appears seriously damaged as in 4c.

In the second line of Fig. 4(d–f), a strong cytotoxic effect of MoS_2_ nanosheets is revealed on U937 cells, resulting in a massive cell death of this hematological cellular system. 4d shows the typical appearance of a control U937 cell (in absence of MoS_2_ dispersion). The cell death is associated to deposition of sodium chloride crystals appearing both as little cubes and massive aggregates. In Fig. 4 In 4e, treated U937 cells are heavily damaged as compared to the control. The presence of 2D nanosheets induced a complete distortion of the U937 cell structure upon their interaction, leading to complete cell death. Both sodium chloride types of crystals are visible as typical final product of cell decomposition induced by the stress due to MoS_2_ nanoflakes reported in 4 f. In this case a fragment of decomposed U937 cell is shown, surrounded by the sodium chloride crystals that probably coat also the smaller MoS_2_ flakes.

In the last line of Fig. 4(g,h), untreated HaCaT cells represent a mesh-like structure with several filaments over the periphery of its cell membrane in 4g. MoS_2_ nanosheets treatment at 10 *μg*/*mL* caused a disruption of the cell structure with separation of the mesh into two parts. The presence of 2D flakes, highlighted in the figure by red arrows, resulted in destruction of the HaCaT cell membrane in 4h. MoS_2_ dispersion at 14 *μg*/*mL* shows the presence of 2D multilayer flakes over the cell surface, where craters also appear in the membrane as a result of the mechanical damage induced by the MoS_2_ nanosheets to HaCaT cells in 4i.

Taken together, these results provide evidences of the capability of MoS_2_ nanosheets to interact with the cellular surface and to trigger changes in cell morphology that likely evidence a strong mechanical damage.

### MoS_2_ nanosheets and cells interaction pathway

**Figure 5:**
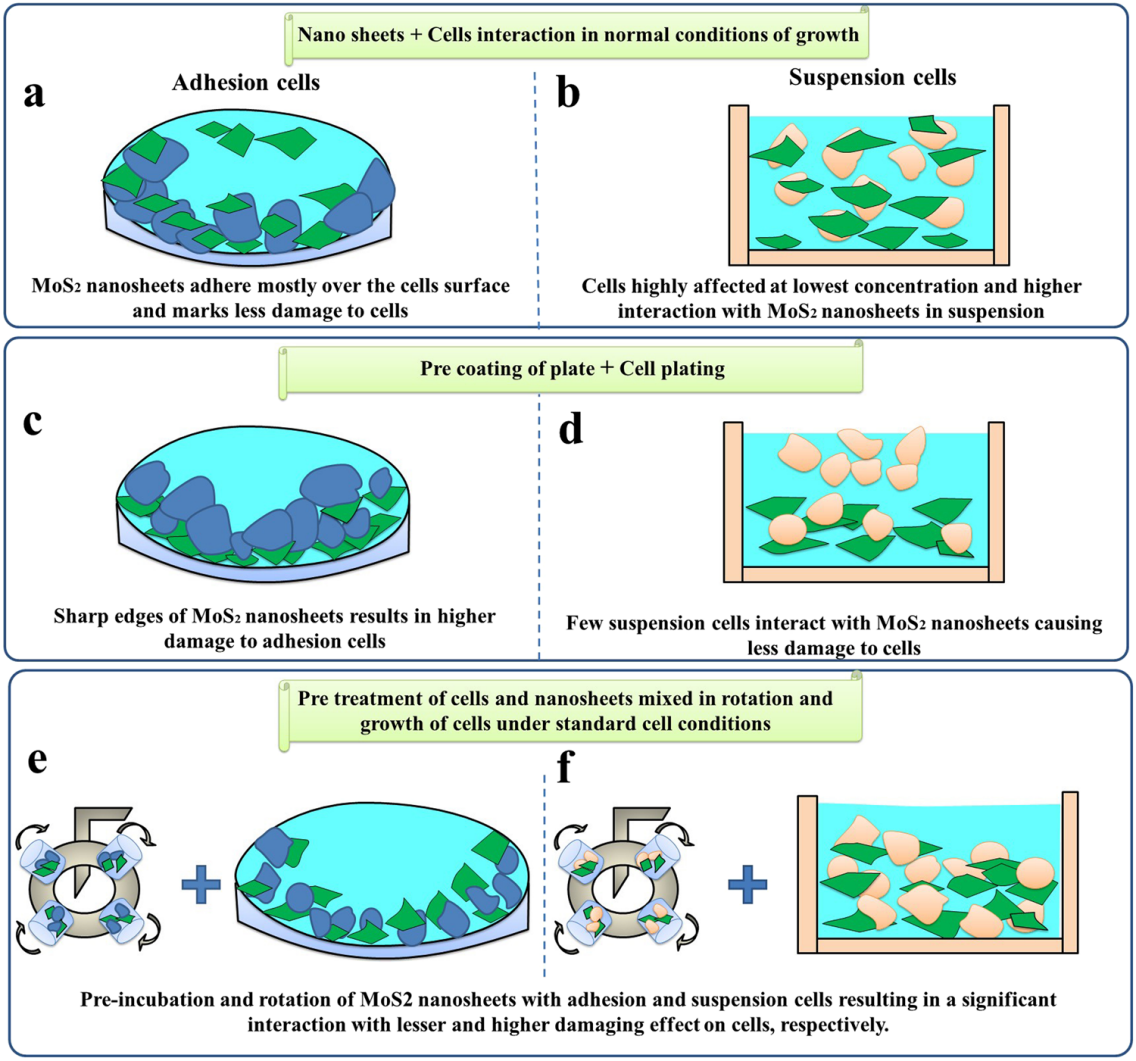
Interaction pathway for adhesion (MCF7 and HaCaT cells) and suspension cells (U937) with 2D MoS_2_ nanosheets.

Figure 5 explains a simple scheme of three different interaction ways of 2D MoS_2_ nanosheets with adhesion and suspension human cells. Figure 5a shows the adhesion interaction of MoS_2_ nanosheets over the cell surface. In such a case, MCF7 cells (adherent) were coated over the cell plate and then MoS_2_ dispersion was added for 72 h incubation. The cell viability was not affected even after 48 h of exposure with nanosheets. We observed a change in S phase of cells after 72 h of incubation because of slow sedimentation velocity of MoS_2_ nanosheets in cell medium (Fig. 2). A Similar effect was observed in case of HaCaT cells (again adherent). In Fig. 5b, the interaction scheme of MoS_2_ nanosheets with suspension cells (U937) is represented. After 48 h incubation with nanosheets, time dependent cell death was observed together with a high decrease in cell viability even at the lowest concentration of the MoS_2_ dispersion (in Fig. 2).

In Fig. 5c, MoS_2_ nanosheets dispersion was coated over the plates and then cells were exposed to the nanosheets surface. In case of adhesion cells (MCF7), after 24 h incubation, more than 50% cell death was observed at the lowest cell density because a better contact between flakes and cells takes place resulting in maximum damage (Fig. 3). In Fig. 5d, suspension cells exhibit less damage because of the smaller chance to interact with the coated plated surface treated with MoS_2_ nanosheets (Fig. 3).

**Figure 6:**
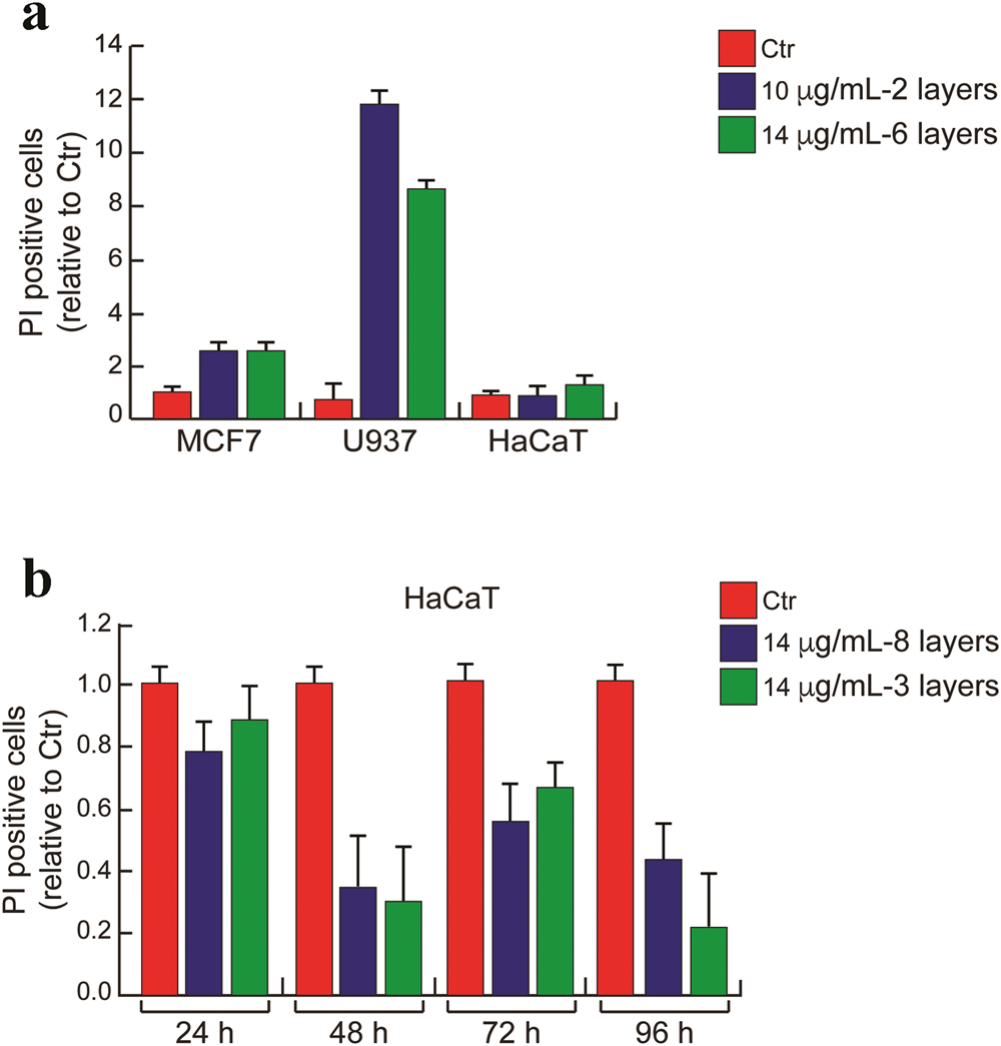
Cell death analysis for long duration. (**a**) Cell Death induced by MoS_2_ nanosheets treatment in the same experimental setting of SEM experiment. (**b**) Cell Death induced by MoS_2_ nanosheets treatment with HaCaT cells checked at 24, 48, 72 and 96 h after treatment, in the same experimental conditions of SEM experiment. Error bars report standard deviation after three independent experiments in (**a**,**b**).

In Fig. 5e, MoS_2_ dispersion and cells were pre-incubated and rotated for 1 h to have better mixing and maximum interaction in the dispersion. In this respect, we observed the exciting result of maximum damage to both the tumoral cell lines and negligible effect to the normal one. This interaction between cells and MoS_2_ nanosheets resulted in more virulent nature for the cells already in suspension (U937). While in case of adhesion cells, as they adhere slowly to the bottom of the plate, their interaction resulted in a minor less damage for HaCaT cells (normal) compared to tumor MCF7 in Fig. 6a.

### MoS_2_ mediated cell death evaluation

We integrated the qualitative morphological analysis of the MoS_2_ nanosheets impact onto the above mentioned three cell lines, carried out by SEM images, by evaluating the cell death based on FACS. Then, a quantitative estimate of the induced cell death after 24 h is obtained as percentage of cells positive to the Propidium Iodide test, as shown in Fig. 6a. The result is very surprising and somewhat striking: while MoS_2_ nanosheets were able to induce cell death in both of the cancer cell lines, they essentially did not in normal cell line, as shown in Fig. 6, where the Propidium Iodide positive cell level is even lower than the untreated control indicating no induction of cell death. In fact, in breast cancer MCF7 cell line, MoS_2_ dispersion incubated in both quantities induced a two fold increase of cell death. Acute myeloid leukemia U937 cell line appeared the most sensitive to 2D nanomaterial treatment, with an increase of cell death of 8–12 folds as compared to the untreated cells. Negligible effect was instead observed in HaCaT cell line even when these cells were exposed to MoS_2_ nanosheets for longer duration of observation up to 96 h after treatment: a difference in favor of the untreated cells was observed here, demonstrating the inefficacy of MoS_2_ nanosheets in such cell system in Fig. 6b. Interestingly, the anti-proliferative effect is obtained both in hematological and solid cancer cell lines, appearing at this stage to be a cell-type-independent cancer response, restricted to the only tumor cells. HaCaT cells in fact, here utilized as a model for non-cancer cells, are unaffected in each scheme of treatment (Fig. 5) but the case where MoS_2_ nanosheets dispersion was coated over the plates and then cells were exposed to the nanosheets surface in Fig. 5c, which resulted in a weaker effect as compared to the analog for tumor cells see Supplementary Table S4 and Fig. 3(a–d). Whether this feature is a general finding characterizing cancer cells regardless their type or is cell-type-dependent, for instance because of the interaction between the negatively charged nanosheets and the cell membrane having an electrostatic potential varying from type to type, is a very intriguing question, whose response to is out of the scope of this paper and will be addressed in further investigations. This finding indicates MoS_2_ nanosheets as a possible promising atoxic tool in cancer therapy. If confirmed this preliminary observation would be of extreme importance, and would open the route to concrete applications of MoS_2_ nanosheet treatment in living systems as possible targeted anti-cancer system. It is worth pointing out that this result is not at odd with morphological analysis based on SEM investigation of the treated cells, that indicated possible mechanical damage in all the three cell lines, since morphological analysis is not quantitative and basically enlightens only mechanical stresses.

## Antibacterial effect by naked MoS_2_ nanosheets

Graphene and its derivatives have been explored a lot for their antibacterial activities, but there are few studies which reflect the antibacterial mechanism of TMDs. Liu. X. *et al*. studied time and concentration dependent antibacterial activity of WS_2_ nanosheets on gram negative *E. coli* and gram positive *Staphylococcus aureus* bacteria^68^. Shinde and co-workers demonstrated the inhibitory effects of WS_2_ and WS_2_-rGO composite nanosheets on gram negative (*E. coli*) and *Salmonella typhimurium (S. typhimurium)*, and Gram positive *Bacillus subtilis (B. subtilis)* and *Staphylococcus epidermidis (S. epidermidis)* bacterial strains^48^. Na Wu *et al*. studied the toxicity of MoS_2_ on *E. coli* with its increasing concentration by utilizing metabolomics technology^44^. Studies on *Salmonella* bacteria using TMDs have generally been explored very little. Zhang X. *et al*. studied chitosan functionalized and antibiotic loaded MoS_2_ nanosheets to combat the *S. aureus* and gram negative *Salmonella* bacteria against the bacterial resistance and biofilm formation^42^.

To utilize 2D TMDs in various antibacterial applications, it is important to study first the interaction of 2D nanosheets with the bacterial target. In this aspect, we have chosen *S. typhimurium* which is well known to be a pathogen causing nosocomial infections, often contaminating water or food^69^. The latter case is very much timely: a number of applications are currently investigated one of the most interesting examples being the possibility to make new food packaging systems to avoid or to reduce *Salmonella* contamination of food. The two different categories of *Salmonella* bacteria (ATCC 14028 and wild-type (WT) *Salmonella*) were used to interact with liquid exfoliated MoS_2_ nanosheets. A clear antibacterial action of MoS_2_ nanosheets was observed in SEM images where MoS_2_ nanosheets acted as a sharp knife and cuts the outer membrane of *Salmonella*. Then MTT assay was performed to study the bacterial viability after the interaction with MoS_2_ nanosheets.

### Antibacterial activity: proliferation test

Laboratory prepared *Salmonella* ATCC 14028 and wild-type (WT) *Salmonella* were used as representative bacterial strains to study the bactericidal activities of MoS_2_ nanosheets.

**Figure 7:**
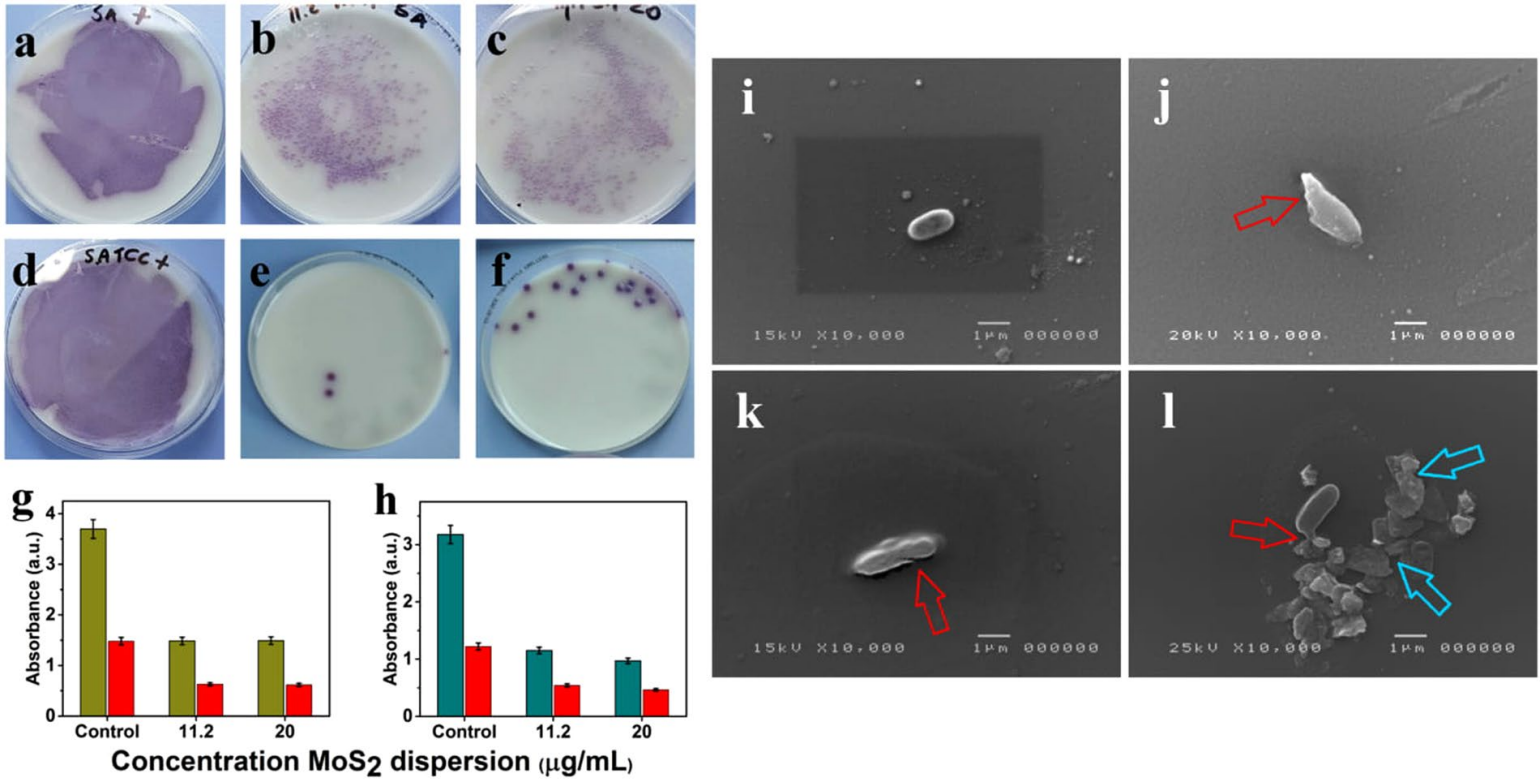
Proliferation test. Two different concentrations of 2D MoS_2_ dispersion interacted with *S. typhimurium* represented as (**a**–**f**). (**a**) Positive control of wild-type *Salmonella* without 2D MoS_2_, (**b**) incubation at 11.2 *μg*/*mL* and C) at 20 *μg*/*mL*; (**d**–**f**) *Salmonella* bacteria grown in the lab (ATCC 14028), (**d**) positive control without 2D MoS_2_, (**e**) incubation at 11.2 *μg*/*mL* and (**f**) at 20 *μg*/*mL*. All the samples treated with MoS_2_ nanoflakes were checked after 4 h from the treatment. (**g**) MTT plots of 2D MoS_2_ dispersion incubated with ATCC 14028 *Laboratory Salmonella* and (**h**) wild-type *Salmonella* at 11.2 *μg*/*mL* and 20 *μg*/*mL*. The absorbance of the incubation is presented at 450 nm (green) and 490 nm (red) wavelengths. Interaction of 2D MoS_2_ nanosheets at 20 *μg*/*mL* with average thickness of $\bar{N}$ = 4. (**i**) Control rod shaped ATCC 14028*S. typhimurium* (**j**) fragment of a bacterium cut by a nanosheet evidenced by the red arrow, (**k**) slight cut at the outer bacterial membrane as shown by red red arrow, and (**l**) leakage of intracellular components of *Salmonella* bacteria (red arrow) upon interaction with the sharp edges of the 2D MoS_2_ nanosheets present nearby (blue arrows).

Incubation of *S. typhimurium* bacterial models was performed at two different concentrations of 2D MoS_2_ nanosheets dispersion (11.2 and 20 *μg*/*mL*) for 24 h. Bacterial viability was studied via a proliferation test. Samples incubated with MoS_2_ nanosheets dispersion (properties of MoS_2_ nanosheets dispersion mentioned in Supplementary Table S3) were checked after the first four hours from the treatment, and then re-checked after 24 h to investigate the bacterial death (Fig. 7). Incubation of the bacterial models under the same conditions were used as a positive control for the experiment, without MoS_2_ nanosheets dispersion. From the colony counting images (Fig. 7) we can clearly see an antibacterial effect in all cases. The antibacterial action of MoS_2_ nanosheets is due to both membranes mechanical injury, as imaged in Fig. 7(i–l) by SEM, and oxidative stress^70^. From Fig. 7(a–f), we observed a clear antibacterial effect of 2D MoS_2_ nanosheets on *S. typhimurium*. In 7a, SA+ denotes the control wild-type *Salmonella* with no incubation with 2D nanosheets The bactericidal action is very clear and similar in 7b at 11.2 *μg*/*mL* with $\bar{N}$ = 2 and in 3c at 20 *μg*/*mL* with $\bar{N}$ = 4. From Fig. 7(d–f), we observed an even more evident bactericidal effect on the ATCC 14028 *Salmonella*, which resulted in complete death of bacterial cells upon incubation with sharp edged 2D MoS_2_ nanosheets. In 7d, SATCC+ denotes the positive control ATCC 14028 lab *Salmonella typhimurium* other than the wild-type. Bactericide effect in ATCC 14028 *Salmonella* is much stronger than the corresponding case in wild-type, as somewhat expected. Moreover, differences between the two concentrations of the nanoflakes as shown in 7e and 7f can be considered negligible, being the number of counted colonies in the range of some units in both cases. We interpret our finding as, the MoS_2_ flakes could act as nano-knives or nano-blades on the *Salmonella* bacteria, being capable to cut the bacterial external cell wall since the flake has a smaller or approximately the same thickness as the wall, this latter being 10–12 nm^71^. Alternatively, sheets can wrap around the cell surface (wrapping) without penetrating it. In a further mechanism, called trapping, a net of MoS_2_ flakes traps bacteria. This will be strikingly clear from SEM images of the bacteria treated with MoS_2_ nanosheets in the forthcoming section.

## MTT assay

### MTT plots of lab prepared Salmonella incubation with 2D MoS_2_ dispersion

In Fig. 7(g,h), we have demonstrated the effect of incubation of two different $\bar{C}$ of 2D MoS_2_ nanosheets with ATCC 14028 and wild-type *S. typhimurium*. MTT analysis reveals the oxidative stress generated upon incubation of MoS_2_ nanosheets with bacteria. From Fig. 7g,h we can see that for both *Salmonella* types a relevant oxidative stress was induced, reducing the absorbance to about 40% of the untreated sample.

## Scanning Electron Microscopy measurement (SEM)

### Antibacterial activity: the action mechanisms of MoS_2_ nanosheets

In Fig. 7(i–l), we strikingly observe the role of direct contact of 2D MoS_2_ nanosheets, having sharp edges, with the bacterial membrane. In 7i, under the control experiment we can clearly see the normal rod shaped morphology with smooth and intact membranes of a ATCC 14028*S. typhimurium*. After incubating with 2D MoS_2_ dispersion, a stress in the membrane is visible, that causes bacteria fragmentation as in 7j and/or cuts in the bacteria membrane as in 7k, where we can figure out the action of the 2D MoS_2_ nanosheets sharp edges as they acted as a sharp nano-knife. In 7l, leakage of the intracellular components results in complete distortion of the bacterial membrane caused by mechanical stress induced by the nanoflakes laying nearby.

## Conclusions and Future prospects

We have reported a novel green route for scalable production of defect-free and few-layered MoS_2_ nanosheets by direct exfoliation in pure water. Exfoliation of MoS_2_ nanosheets using water as a solvent via LPE is a big challenge itself. Therefore, after optimizing the most relevant parameters for exfoliation, we achieved a stable dispersion for up to three weeks. Additionally, by using different centrifugal forces we attained size and thickness selection of nanosheets possibly restricting our production in the 2–5 layer band. Samples were characterized by absorption measurements which revealed the final mean concentration of the dispersion, mean lateral size and mean thickness of MoS_2_ nanoflakes. *ζ*- potential measurements estimated the negative surface potential of MoS_2_ nanosheets. Interactions of few-layered MoS_2_ nanosheets on live human cells and bacteria were also investigated. Here, we found a very interesting and novel result: the impact of MoS_2_ nanoflakes was found to be quite different in normal from cancer cell lines. While the latter cells revealed a significant cytotoxic effect based on a very large increase of cell death, the former were essentially unaffected in this respect and only showed some mechanical damage when morphologically analyzed by SEM microscopy. This cytotoxic effect was also found to dependent on the concentration and layer number of 2D nanoflakes. In the near future, this preliminary analysis might open up new routes for significant applications of MoS_2_ nanosheets as targeted anti-cancer systems. This analysis was further extended to bacteria. SEM images of *S. typhimurium* treated with 2D nanosheets revealed that the sharp edges of the nanoflakes can cut and/or damage bacterial membrane leading to an evident bactericidal effect. Bacterial viability was studied by colony counting images and MTT assay that probed possible oxidative stresses induced by treatment with the nanoflakes. The results obtained by treating *Salmonella* bacteria with MoS_2_ nanosheets are interesting and will be further extended to higher concentrations of 2D MoS_2_ dispersions. One might see whether the interaction with nanoflakes in such condition leads to an increase of intra-cellular metabolites or might investigate the effects on amino acids and pyruvate metabolism. This could help in clarifying the mechanism of the antimicrobial effect of MoS_2_ nanosheets. The results obtained in studying the impact of water-based preparation of MoS_2_ few-layered nanoflakes with live matter represent an important step to unveil the scenario of the interactions of these novel materials with bacteria, viruses and human cells. Moreover, further optimization of a number of parameters in the exfoliation can additionally improve the quality of the nano-samples in terms of biocompatibility and stability of the water-based dispersion. This is essential from a practical point of view aiming at designing and realizing applications of this innovative 2DMs to biomedical sciences and food packaging. This innovative preparation technique is versatile and can be easily extended to other 2DMs at large scale production.

## Materials and Methods

### Exfoliation of MoS_2_ powder

The starting commercialized bulk MoS_2_ powder (Sigma Aldrich, 69860, particle size 6 *μm*, density 5.06 g *mL*^−1^ at 25 °C) was exfoliated in elix water as a pure solvent using a tip sonicator (Bandelin Ultrasound SONOPLUS HD3200, maximum power 200 W, working frequency 20 kHz, KE-76 probe, running at 15% amplitude) for 3 h in cylindrical glass tubes (2 cm diameter, 12 cm height and rounded bottom). The temperature of the dispersion during sonication was controlled in an ice-water bath. Successive stepwise controlled centrifugation steps were carried out (Eppendorf Centrifuge 5810 R, Rotor F-34-6-38) at 40 g, 160 g, 620 g and 1000 g/1400 g for 45 minutes each to analyze the supernatants.

### Controlled centrifugation

Liquid phase exfoliation (LPE) produces broad distribution of thickness and lateral sizes of nanosheets. The polydisperse behaviour of the exfoliated dispersion makes it desirable to study its potential for fundamental applications in various areas of interest. Controlled centrifugation by optimizing its parameters (such as centrifugation speed, centrifugation time, rotation angle, inside temperature, acceleration and deceleration values) is performed to achieve the size selection^21^. Execution of controlled centrifugation is very versatile and can be achieved by benchtop centrifuges. In our experiment protocol, the un-exfoliated nanosheets were removed by low centrifugal force at 40 g for 45 minutes. The remaining supernatant contains less monolayer content with wide distribution of thickness and lateral sizes. The supernatant was then centrifuged to a higher centrifugal force of 160 g for same time and the sediment was discarded. The obtained supernatant was again centrifuged with a higher centrifugal force at 620 g for 45 minutes. At this step, we separated half part of the dispersion for basic characterization of the material and the remaining was centrifuged at further higher centrifugal force at 1000 g for 45 minutes each. Then, the final obtained dispersion was utilized to study the properties of MoS_2_. Supplementary Figure S1 shows the UV visible absorption spectra of MoS_2_ nanosheets at 620 g, 2700 g and 3500 g after the immediate preparation and up to three weeks of storage. Higher centrifuged dispersions revealed much improved stability which was achieved by optimizing various parameters after a number of experimental trials. The produced water exfoliated dispersions of MoS_2_ nanosheets, which are highly stable in pure water, are biocompatible and thus can be useful for various biomedical and biotechnological applications.

### Visualization of cell morphology

The HaCaT and MCF7 cells were cultured directly on coverslips. U937 cells were coated on polylisined coverlips before the SEM procedures. The cells were fixed with 2.5% glutaraldehyde in 0.2 M PBS at pH 7.2–7.4 for 2–4 h at 4 °C. The cells were then washed three times with PBS 0.2 M for 10 minutes. Additional fixing was performed by OsO_4_ 1–2% in PBS 0.2 M at pH 7.4 for 2 h at 4 °C in dark. The cells were then washed with PBS 0.2 M (3% for 10 minutes) at 4 °C. The samples were dehydrated by EtOH 30%; 50%; 70%; 80%; 95% for 10 minutes and 100% for 1 h at 4 °C. Morphological analyses of samples were performed with a scanning electron microscope (SEM) JEOL-JSM 5310 (CISAG laboratory, at University of Naples, Federico II). The SEM operating at 15 kV, is equipped with energy dispersive X-Ray spectroscopy (EDS); data were processed with INCA version 4.08 (Oxford Instruments, 2006). The samples were metalized with gold by using a sputter coater. Oxford Instruments (2006): INCA - The microanalysis suite issue 17a + SP1 - Version 4.08. Oxford Instr. Anal. Ltd., Oxfordshire, UK.

### Cell line culture, conditions and preparations

U937 (acute myeloid leukemia cell line) cells were grown in RPMI 1640 medium (EuroClone) supplemented with 10% heat-inactivated FBS (Sigma Aldrich), 1% glutamine (EuroClone), 1% penicillin/streptomycin (EuroClone) and 0.1% gentamycin (EuroClone), at 37 °C in air containing 5% CO_2_. MCF7 (human breast adenocarcinoma cell line) and HaCaT (immortalized non tumorigenic human keratinocytes) cells were grown DMEM medium supplemented with the same components described above and in the same incubation conditions.

### MTT- Cell proliferation assay

The MoS_2_ nanosheets were dispersed in Elix water at different concentrations (8, 14 and 20 *μg*/*mL*). Dilutions by a factor of about four as compared to the concentration value of the initial preparation were performed. The cell viability was evaluated using 3-[4, 5-dimethyltriazol-2-yl]-2, 5-diphenyl tetrazolium bromide (MTT) as substrate. MTT assay (Sigma Aldrich) was performed according to the protocol provided by Supplier. The absorbance was measured with microplate reader (Tecan EVO M1000 PRO) at the wavelength of 570 nm and using 630 nm as reference wavelength. Experiments were performed in triplicate.

### Propidium iodide staining: cell death evaluation

After the induction with MoS_2_ for different times and at different concentrations, cells were collected and centrifuged at 1200 rpm and then washed with cold PBS. Cell pellets were re-suspended in PI staining solution (0.2 *μg*/*mL*). PI positive cells were counted by flow cytometry (FACS). Experiments were performed in triplicate.

### Cell cycle analysis

Cells were collected by centrifugation at 1200 rpm for 5 minutes and then re-suspended in 500 *μL* of a hypotonic buffer composed of 0.1% NP-40, 0.1% sodium citrate, 50 *μg*/*mL* propidium iodide (Sigma Aldrich), RNAse A. The samples were then incubated in the dark for 30 minutes. Analysis was performed by FACS-Calibur (Becton Dickinson) using Cell Quest Pro software (Becton Dickinson) and ModFit LT version 3 software (Verity). Experiments were performed in triplicate.

### Microbial strains, culture conditions and preparations

We used *(S. typhimurium*) ATCC 14028 and wild-type *S. typhimurium* as a model bacterium to evaluate the antibacterial activity of MoS_2_ nanosheets. Also all the bacterial samples without the incubation of nanosheets were used as a positive control in nuclease free water. The bacterial cell suspension was diluted in isotonic saline solution to obtain cell samples containing 150 colony forming units (CFU). Cell growth was determined by measuring the optical density at 600 nm (Lambda-25 spectrophotometer, Perkin-Elmer, USA) in six parallel measurements for each time-point. *S. typhimurium* ATCC 14028 and wild-type *S. typhimurium* were maintained on buffered peptone water (BPW) at 37 °C under constant orbital shaking at 220 rpm for up to 24 h. The MoS_2_ nanosheets dispersion was diluted at two different concentrations 11.2 *μg*/*mL* and 20 *μg*/*mL*, respectively, using culture medium with a final concentration of bacteria of 1 × 10^6^ CFU *mL*^−1^. Both categories of *S. typhimurium* were cultured at the condition of 37 °C for up to 6 h. Antibacterial effect was evaluated by the colony counting method. In brief, the incubation bacterial solutions were initially diluted to 1 × 10^5^ CFU *mL*^−1^. Later, 100 *μL* of the diluted bacterial cells were spread respectively on the *Salmonella* Chromogen Agar plates. After incubation overnight at 37 °C for *S. typhimurium*, colonies on the plates were counted and compared with those on the control plates (without any MoS_2_ nanosheets) to calculate the loss of viability caused by the MoS_2_ nanosheets samples.

### Bacterial cell growth

Bacteria were diluted up to 10^6^ CFU/mL and exposed to MoS_2_ nanosheets at different concentrations in a final volume of 100 *μL*. Experiments were made in duplicates. Different final concentrations of MoS_2_ were tested at 11.2 *μg*/*mL* and 20 *μg*/*mL*. Aliquots were collected after four hours, conveniently diluted by serial dilutions 1:10 and plated in *Salmonella* Chromogen Agar plates. The plates were incubated overnight at 37 °C. CFU were counted the following day.

### Determination of bacterial viability

Methylthiazolyldiphenyl-tetrazolium bromide (MTT) reagent (Sigma-Aldrich,USA) was used for bacterial viability measurements. Two different categories of *Salmonella* bacteria were exposed to 20 *μg*/*mL* and 11.2 *μg*/*mL* of MoS_2_ nanosheets in PBS. 10 *μL* of the 12 mM MTT stock solution was added to each well. A negative control of 10 *μL* of the MTT stock solution was added to 100 *μL* of medium alone. Then this solution was incubated at 37 °C for 4 h. At high cell densities the incubation time can be shortened to 2 h. Then, 100 *μL* of the SDS-HCl solution was added to each well and thoroughly mixed using the pipette. Then, the microplate was incubaed at 37 °C for 4 h in a humidified chamber. Longer incubations generally decrease the sensitivity of the assay so short incubation time was more preferred in this experiment. After mixing each sample again using a pipette the absorbance was observed at 450 and 490 nm.

### Visualization of bacterial morphology

Changes in the morphology of salmonella bacteria were studied using scanning electron microscopy (SEM). Obtaining acceptable SEM images with good ultrastructural preservation requires careful application of the SEM sample preparation methods. The concentration of 2D MoS_2_ dispersion and its incubation with *Salmonella* for SEM analysis was chosen at 20 *μg*/*mL*).

After incubation overnight at 37 °C for *S. typhimurium* with/without MoS_2_ nanosheets in buffered peptone water (BPW) for 24 h, preparation for SEM was carried out according to the following protocol:

1. Bacterial broth was centrifuged.
2. Pellet was washed with saline phosphate buffer for 3 times.
3. 0.25% gluteraldehyde was added in sodium phosphate at pH- 7.2.
4. Then this mixture was incubated at room temperature for 30 minutes.
5. Then the overnight incubation was performed.
6. Sodium phosphate buffer was washed for 3 times.
7. After centrifugation, the pellet was collected.
8. The sample was dehydrolysed by different ethanol volumes starting from 30%, 50%, 70%, 80%, 90% and 100%.
9. For each ethanol volume the sample was incubated for 10 minutes.
10. Additionally, incubation of the sample was performed in 100% ethanol volume for 1 h.
11. Sample preparation for SEM was performed by applying adhesive tape and then the bacterial sample was added over the adhesive tape.

## Electronic supplementary material

Supplementary Information
