# Supplementary material for: Biological interactions of biocompatible and water-dispersed MoS2 nanosheets with bacteria and human cells
Source: Sci Rep. 2018 Nov 6;8:16386. doi: 10.1038/s41598-018-34679-y (PMC6219585; doi:10.1038/s41598-018-34679-y)
Supplement: Supplementary file 1 — Supplementary Information [file 41598_2018_34679_MOESM1_ESM.docx]

**Electronic Supplementary Information (ESI)**

**Biological interactions of biocompatible and water-dispersed MoS**_2_ **nanosheets with bacteria and human cells**

**Jasneet Kaur**^1,2,^**^+^, Manjot Singh**^1,^**^+^, Carmela Dell‘Aversana**^3,^**^+^, Rosaria Benedetti**^3,^**^+^, Paola Giardina**^2^**, Manuela Rossi**^4^**, Mohammadhassan Valadan**^1^**, Alessandro Vergara**^2^**, Anna Cutarelli**^5^**, Angela Michela Immacolata Montone**^5^**, Lucia Altucci**^3^**, Federica Corrado**^5,*^**, Angela Nebbioso**^3,*^**, and Carlo Altucci**^1,*^

^1^Department of Physics, “Ettore Pancini”, University of Naples “Federico II”, Naples, Italy.

^2^Department of Chemical Sciences, University of Naples “Federico II”, Naples, Italy

^3^Department of Precision Medicine, University of Campania “L Vanvitelli, Vico L. De Crecchio” 7, 80138, Naples, Italy

^4^Department of Earth, Environment and Resources Sciences, University of Naples “Federico II”, Naples, Italy

**^5^**Experimental Zooprophylactic Institute of Southern Italy, Portici, Italy

^*^Email: [altucci@na.infn.it](file:///C:\Users\Manjot\Downloads\altucci@na.infn.it), [angela.nebbioso@unicampania](mailto:angela.nebbioso@unicampania).it and [federica.corrado@cert.izsmportici.it](mailto:federica.corrado@cert.izsmportici.it)

^+^These authors contributed equally to this work.

***Characterization tools***

***UV-visible spectrum***

Optical extinction spectra were acquired on Jasco V-530 UV-Vis spectrophotometer using 1 cm optics quartz cuvettes. The extinction spectra of MoS_2_ dispersion was analyzed to determine the Mean layer number (*N*), Mean lateral size (*L*) and Mean concentration (*C*) of the nanosheets by metrics as explained by Backes et al. ^1^

**

**

**Figure S1. UV-visible extinction spectrum of MoS_2_ nanosheets dispersion at 620g, 2700g and 3500g.**

***Zeta potential measurements***

Electrostatic stabilization is an important parameter to analyze the stability of the liquid exfoliated dispersions. The surface charges generated during the exfoliation can be attributed to electrophoretic mobility measurements (µ). So, these (µ) measurements were carried out on laser interferometric technique (Malvern Zetasizer Nano system) with irradiation from 633 nm He-Ne laser. The samples were injected in folded capillary cells, and the electrophoretic mobility (µ) was measured using a combination of electrophoresis and laser Doppler velocimetry techniques. ^2^ The Henry’s equation was used to estimate the zeta potential from the electrophoretic mobility data. For the possible upper and lower limits of the zeta potential values, Henrys equation was approximated to both the Huckel and Smoluchowsky limits. The reason for this approximation is due to the particular solvent-sample relationship; Henrys equation is approximated to get an estimate of surface charge values of exfoliated nanosheets. All the measurements were carried out at 25 ºC.

***Raman and Photoluminescence spectroscopy***

A confocal Raman microscope (Jasco, NRS-3100) was used to obtain Raman and photoluminescence spectra. The 514 nm line of an air-cooled Ar+ laser (Melles Griot, 35 LAP431 220), was injected into an integrated Olympus microscope and focused to a spot diameter of approximately 3 μm by a 20x objective with a final 4 mW power at the sample. A holographic notch filter was used to reject the excitation laser line. The Raman backscattering was collected using a 0.1 mm slit and a diffraction lattice of 1200 grooves/mm, corresponding to an average spectral resolution of 8 cm**^-^**^1^. Solutions were left evaporating on Si substrates, and it took 60 s to collect a complete data set by a Peltier-cooled 1024 x 128 pixel CCD photon detector (Andor DU401BVI). Raman measurements were at least triplicated for scope of reproducibility. Wavelength calibration was performed by using cyclohexane as a standard.

| **S. No.** | **Centrifugal Force** | **Mean Layer Number**  **(N)** | **Mean Lateral Size**  **(L)** |
| --- | --- | --- | --- |
| 1.  2.  3. | 620g  1000g  1400g | 5  2-3  2 | 220 nm  160 nm  100 nm |
| 4.  5. | 2700g  3500g | 3  2 | 90 nm  70 nm |

**Table S1. Parameters achieved after the exfoliation of bulk MoS_2_ via different centrifugal forces.**

| **Parameters achieved after the exfoliation** | **Values of the parameters**  **after the exfoliation** | **Values of the parameters after three weeks of storage** |
| --- | --- | --- |
| A - exciton value | 662 nm | 662 nm |
| Number of layers (***N*)** | 2 | 2 |
| Concentration (µg/mL) (***C*)** | 10 µg/ml | 10 µg/ml |
| Lateral size (***L*)** | 76 nm | 71 nm |

**Table S2. Extinction spectrum parameters just after exfoliation and after three weeks of storage.**

| **Concentration of MoS**_2_ **dispersion** | **Layer Number**  **(N)** | **Lateral Size**  **(L)** |
| --- | --- | --- |
| **5** µg/mL  **2** µg/mL  **0.5** µg/mL | **9**  **6**  **5** | **220 nm**  **180 nm**  **160 nm** |

**Table S3. Properties of exfoliated MoS_2_ nanosheets used in cell cycle progression experiments.**

| **Concentration of MoS_2_ dispersion**  **(µg/mL)** | **Layer Number**  **(N)** | **Lateral Size**  **(L)** | **Absorbance after**  **24 h** | **Absorbance after**  **48 h** |
| --- | --- | --- | --- | --- |
| 10  14  14 | 2  10  6 | 145 nm  250 nm  220 nm | 0.55 ± 0.02  0.54 ± 0.02  0.63 ± 0.02 | 0.57 ± 0.06  0.45 ± 0.02  0.57 ± 0.01 |
| 22  36 | 5  12 | 200 nm  300 nm | 0.61 ± 0.01  0.64 ± 0.01 | 0.53 ± 0.01  0.49 ± 0.01 |

**Table S4. Concentration, mean layer number and mean lateral size of exfoliated MoS_2_ nanosheets pre-incubated with 3000 HaCaT cells for 24 h and 48 h. Absorbance values after 24 h and 48 h of incubation with nanosheets. Negative control is normalized to absorbance equal to 1.**

| **Concentration of MoS_2_ dispersion**  **(µg/mL)** | **Layer Number**  **(N)** | **Lateral Size**  **(L)** | **Absorbance after**  **24 h** | **Absorbance after**  **48 h** |
| --- | --- | --- | --- | --- |
| 10  14  14 | 2  10  6 | 145 nm  250 nm  220 nm | 0.163 ± 0.012  0.118 ± 0.002  0.120 ± 0.002 | 0.194 ± 0.002  0.136± 0.001  0.107± 0.001 |
| 22  36 | 5  12 | 200 nm  300 nm | 0.299 ± 0.003  0.209 ± 0.012 | 0.358± 0.003  0.221 ± 0.012 |

**Table S5. Concentration, mean layer number and mean lateral size of exfoliated MoS_2_ nanosheets pre-incubated with 3000 MCF7 cells for 24 h and 48 h. Absorbance values after 24 h and 48 h of incubation with nanosheets. Negative control is normalized to absorbance equal to 1.**

***Yield estimation***

For estimating the process yield of our preparation protocol we carried out the same experiment as referred to in the main text (*See Exfoliation of MoS_2_ powder in Materials and Methods section*). After exfoliation, controlled centrifugation was performed by optimizing its parameters (such as centrifugation speed, centrifugation time, rotation angle, inside temperature, acceleration and deceleration values), to obtain the desired thickness and lateral sizes of nanosheets. While carrying out the centrifugation step, the un-exfoliated nanosheets were removed by low centrifugal force at 40g and 600g. The supernatant was then centrifuged at higher centrifugal force of 1000g and 1400g. The final obtained dispersion was utilized to study the UV-vis absorbance spectra of 2D MoS_2_ nanosheets. Successively, we let samples to dry to achieve the powder form under vacuum evaporation overnight. Then, we weigh the powder and use the following formula^3^ to estimate the final process:

$\alpha=\frac{C_{1}}{C_{0}}\times100\%$

where *α* is the overall yield and *c*_1_ and *c*_0_ are the final and initial concentrations of MoS_2_ nanosheets dispersion, respectively.

The final estimated yield for *c_0_*=5 mg/mL of MoS_2_ nanosheets is ≈ 0.6%. This value is lower than typically obtained in organic solvents and their mixtures, where it is possible to achieve ≈ 2-5%. ^4^ This is due to the solubility of MoS_2_ nanosheets in pure water that is poorer than in organic solvents. However, by adding some biosurfactants or biomolecules such as hydrophobins, more concentrated and more stable MoS_2_ based dispersions can be produced in eco-friendly and biocompatible solvents, which can be useful for multiple applications.

**References**

1. Kaur, J. *et al.* Green synthesis of luminescent and defect-free bio-nanosheets of MoS _2_ : interfacing two-dimensional crystals with hydrophobins. *RSC Adv.* **7,** 22400–22408 (2017).

2. Lotya, M. *et al.* Liquid Phase Production of Graphene by Exfoliation of Graphite in Surfactant / Water Solutions Liquid Phase Production of Graphene by Exfoliation of Graphite in Surfactant / Water Solutions. 3611–3620 (2009). doi:10.1021/ja807449u

3. Dong, H., Chen, D., Wang, K. & Zhang, R. High-Yield Preparation and Electrochemical Properties of Few-Layer MoS2Nanosheets by Exfoliating Natural Molybdenite Powders Directly via a Coupled Ultrasonication-Milling Process. *Nanoscale Res. Lett.* **11,** (2016).

4. Li, Y., Yin, X. & Wu, W. Preparation of Few-Layer MoS2 Nanosheets via an Efficient Shearing Exfoliation Method. *Ind. Eng. Chem. Res.* **57,** 2838–2846 (2018).
